# Long-Range Intra-Protein Communication Can Be Transmitted by Correlated Side-Chain Fluctuations Alone

**DOI:** 10.1371/journal.pcbi.1002168

**Published:** 2011-09-29

**Authors:** Kateri H. DuBay, Jacques P. Bothma, Phillip L. Geissler

**Affiliations:** 1 Department of Chemistry, University of California at Berkeley, Berkeley, California, United States of America; 2 Chemical Sciences, Physical Biosciences, and Materials Sciences Divisions, Lawrence Berkeley National Lab, Berkeley, California, United States of America; 3 Biophysical Graduate Group, University of California at Berkeley, Berkeley, California, United States of America; Harvard University, United States of America

## Abstract

Allosteric regulation is a key component of cellular communication, but the way in which information is passed from one site to another within a folded protein is not often clear. While backbone motions have long been considered essential for long-range information conveyance, side-chain motions have rarely been considered. In this work, we demonstrate their potential utility using Monte Carlo sampling of side-chain torsional angles on a fixed backbone to quantify correlations amongst side-chain inter-rotameric motions. Results indicate that long-range correlations of side-chain fluctuations can arise independently from several different types of interactions: steric repulsions, implicit solvent interactions, or hydrogen bonding and salt-bridge interactions. These robust correlations persist across the entire protein (up to 60 Å in the case of calmodulin) and can propagate long-range changes in side-chain variability in response to single residue perturbations.

## Author Summary

Allosteric regulation occurs when the function of one part of a protein changes in response to a signal recognized by another part of the protein. Such intra-protein communication is essential for many biochemical processes, allowing the cell to adapt its behavior to a dynamic environment. Most studies of the information conveyance underlying allostery have to date focused on the role of backbone motions in mediating large structural changes. Here we focus instead on more subtle contributions, arising from fluctuations of side-chain torsions. Using a model for side-chain bond rotations in the tightly packed environment imposed by native backbone conformations, we observed significant sensitivity of side-chain organization to small, localized perturbations. This susceptibility arises from correlations among side-chain motions that can propagate information within a protein in complex, heterogeneous ways. Specifically, we found appreciable correlations even between side-chains distant from one another, so that the effect of a minor perturbation at one site on the protein could be observed in the altered fluctuations of side-chains throughout the protein. In conclusion, we have demonstrated that the statistical mechanics of correlated side-chain fluctuations within a model of the folded protein provides the basis for an unconventional but potentially important means of allostery.

## Introduction

Allostery is an essential feature of protein regulation and function. Allosteric regulation acts by linking distant sites of a protein together in such a way that information about one site is transmitted to and influences the behavior of another. Chemical modifications as subtle as the phosphorylation of a serine residue can cause dramatic changes in protein function [1], and shifts in structure as small as 1 Å have even been shown to modify behavior in a domain up to 100 Å away [2]. Traditionally, allostery has been understood as a feature of symmetric, multi-subunit proteins where the binding of a ligand to one subunit facilitates the binding of similar ligands to the other subunits, resulting in cooperative binding transitions [3]. However, allosteric behavior has now been observed within a single protein domain [4] and its definition extended to include any shift in protein structure and function at one site resulting from modification at another.

Moreover, it was proposed some time ago that information regarding the binding of a ligand or other modification at one protein site could be transmitted through altered protein fluctuations, even if the protein's average structure remains unaffected [5]. Two particularly clear examples of this kind of dynamic allostery have been recently observed in the binding of cAMP to the CAP dimer and in the subsequent binding of the cAMP-activated CAP dimer to DNA [6], [7]. In the first step, the binding of cAMP to one monomer of CAP lowers the binding affinity of cAMP to the second even though no structural changes are observed, and calorimetric analysis suggests that the negative cooperativity results entirely from entropic effects [6].

The observed allosteric effect of protein fluctuations has led to the idea that allostery may be present in all proteins [8]–[10], and that functional allostery simply exploits and refines pre-existing long-range correlations and interaction networks. In fact, such networks are to be expected given the physical constraints of the densely-folded, yet fluctuating, protein. Just as in any condensed phase, significant fluctuations in this packed environment are permitted through correlated motions.

Qualitative experimental evidence for long-range correlation abounds in studies demonstrating allosteric regulation, as exemplified in [1] and [2]. However, attempts to quantify these long-range correlations using NMR techniques have proven difficult [11]–[13], and much of our current understanding of correlated motions has come from analyses of molecular dynamics (MD) simulations. Traditional MD trajectories evaluated with covariance matrices and principle component analyses [14] have shed light on important features of intra-protein correlations, such as how backbone motions tend to be significantly correlated within secondary structural units [14] and how a few flexible hinge residues can cause large motions within otherwise stable folds [15]. Energy-perturbative MD simulations, such as anisotropic thermal diffusion [16] and pump-probe MD [17], have been used to observe the rapid anisotropic diffusion of an energy perturbation within the protein. However, these MD studies are limited in their ability to characterize sluggish rearrangements and have largely neglected the contributions of correlated side-chain fluctuations.

Within the folded protein, side-chains are significantly less ordered than the backbone [18], and alternative side-chain configurations in protein crystals are more prevalent than previously thought [19]. The thermodynamic importance of this side-chain variability in calmodulin-ligand binding has been highlighted in Refs. [20]–[22]. In addition, the participation of side-chain fluctuations in long-range networks has been demonstrated through NMR mutational studies [9], [23]. In one MD simulation designed to incorporate data from NMR experiments, correlations were even observed between side-chains whose motions appeared decoupled from those of their backbone atoms [24].

Double mutant cycles [25] have also been applied to examine the dependence of folding and binding on interactions between specific residue pairs. While such mutational studies can demonstrate the interactions of certain residue pairs, they are experimentally demanding, making it difficult to obtain a comprehensive picture of any long-range side-chain interactions present, in particular those involving residues essential for folding stability. As an alternative, an evolutionary statistical network analysis method has been developed to determine networks of correlated residues that are common to evolutionarily related proteins [26]. Although this method has had some success in identifying allosterically-related regions within proteins [27], its robustness has been challenged in a study on artificially-generated sequences [28]. In principle, it is also limited to detecting correlated changes in residues during evolution, presumably highlighting only correlated networks with a selected function and can therefore say little about the presence or absence of other correlations.

In this study, we employed an atomistically detailed model to examine the kinds of correlations that emerge among side-chain fluctuations within the natively-folded protein. The computationally inexpensive nature of our model energy function [21], together with a variety of advanced Monte Carlo sampling techniques, allowed an unprecedentedly thorough investigation of the correlations among these fluctuations that result from different types of interactions. Keeping the backbone fixed, we find that long-range correlation of side-chain fluctuations can emerge from different types of atomic interactions, that significant correlations persist across the entire folded protein, and that these correlations alone can propagate changes in structure and mobility over scales as large as 50 Å.

## Results

### A simple model was used to explore side-chain rotations

In order to investigate the correlated rearrangements that arise from side-chain fluctuations alone, it was necessary to isolate these motions from other sources of configurational change. For this reason, we held the backbone fixed in its folded conformation throughout the calculations described in this paper. While fluctuations resulting from bond stretching and angle bending are important and likely to give rise to a great deal of correlated motion, we focused here instead on side-chains' torsional degrees of freedom, as these rotations give rise to the changes in atomic configurations that are largest in magnitude.

This study made use of a model we designed to roughly capture the essential physical determinants of side-chain behavior within the folded protein, namely, steric repulsions, van der Waals attractions, hydrogen bonding, salt-bridge interactions, and solvation [21]. While not fully realistic in every particular (e.g., resolving the positions only of nuclei heavier than hydrogen), the model properly represents the variety, strength, and anisotropy of the side-chain interactions and the physical constraints of the folded backbone on which they reside.

We explored this model with Monte Carlo (MC) sampling (see Methods). Each MC step consisted of the proposed rotation of a single randomly-chosen side-chain dihedral angle. To promote broad sampling of thermally accessible configurations, we permitted moves through sterically disallowed regions of state space. Using exact correction methods, we constructed equilibrium averages with contributions only from sterically allowed structures (those in which each heavy atom excludes a spherical volume with radius 0.75 times its van der Waals radius). See [21] for details.

Boltzmann-weighted ensembles of the side-chain configurations determined using this sampling procedure include a diverse set of rotamer states and correlate well with experimental observations of side-chain fluctuations and changes in entropy upon ligand binding [21]. We therefore applied this method to investigate correlations among the diverse set of rotamer states.

### Single residue perturbations effected changes in side-chain fluctuations throughout the protein

Several experimental approaches that probe correlations within proteins mutate single residues and observe the resulting changes in structure, function, or dynamics [9], [23], [29], [30]. We began our examination of side-chain correlations in a similar way by modeling the changes in torsional variability that occured throughout a small globular protein, barstar, as a result of perturbations to a single side-chain. Previous MD simulations of barstar suggested a relatively rigid backbone, as well as significant variability in side-chain packing [31]. Additional results from 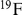 NMR experiments showed that the P27A mutation results in detectable dynamic changes even in residues more than 12 Å away from the mutation site, and suggested that the motion of barstar's side-chains gives rise to a network of correlated residues [32].

Quantifying such correlated fluctuations requires a metric that can report on the extent of local variability at the single residue scale. For this purpose, we calculated the Gibbs entropy for each residue, 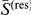, associated with occupying distinct rotameric states.
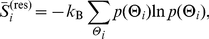
(1)where 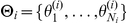 denotes the set of torsional variables for each of the 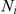 rotatable 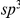-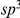 hybridized bonds belonging to residue 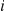, and 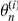 denotes the set of ideal torsional angle values for the 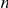
th torsional angle in residue 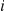. While 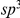-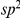 hybridized bonds were allowed to rotate, they were also excluded from the statistical analysis due the difficulty in determining ideal dihedral angles [33]. The probabilities 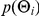 of these 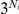 states were calculated in simulations by constructing histograms over the course of importance sampling from the Boltzmann distribution of side-chain configurations. In doing so, we focused on the inter-rotameric rearrangements (those between the three most likely energy basins for the torsional angle of an 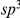-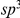 hybridized bond), which allowed the calculation of absolute local entropies that would have been impractical at a higher level of resolution. However, intra-rotameric fluctuations (those within a single torsional energy basin) are sensitive to the structural perturbations we applied, and it is necessary to allow deviations from these ideal angles, 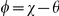, in order to fully account for the variety of possible side-chain configurations [34], [35]. A quadratic energy is associated with these deviations 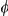 (see Methods).

**Figure 1:**
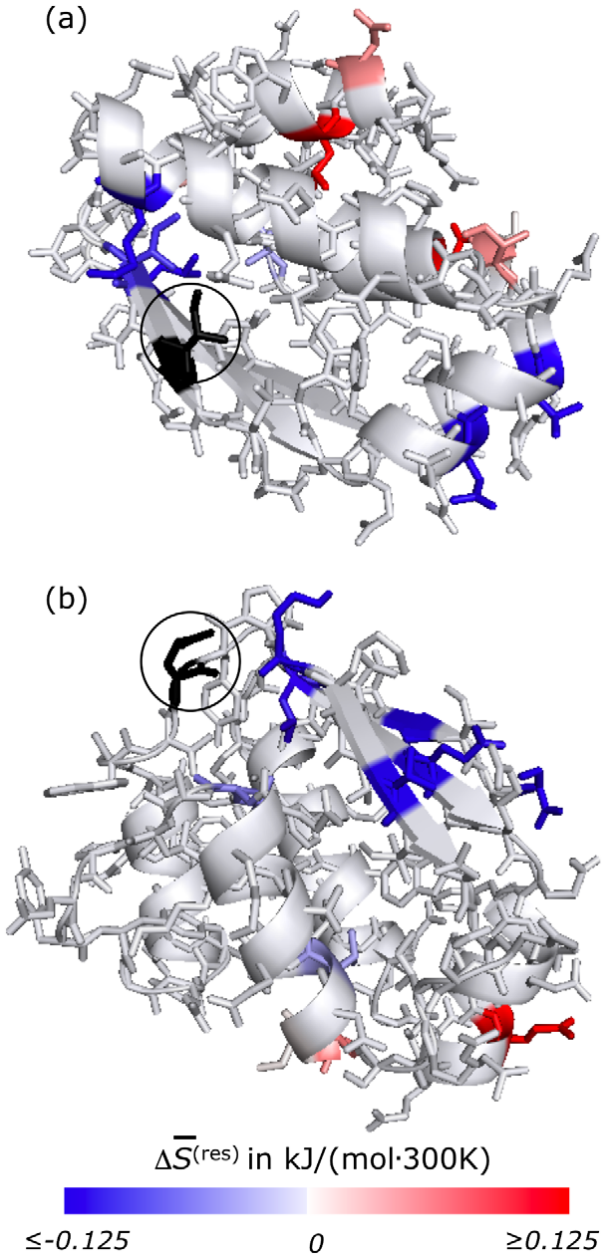
Single-residue perturbations in barstar. Changes 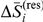 in the Gibbs entropy of each residue 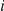 in barstar (1a19 [47]) that resulted from perturbations to single side-chains. Residues whose entropy changes by a significant amount, according to Student's t-test at the 90% level, are shown in color. Red indicates increased entropy, blue indicates decreased entropy (see scale bar). Although side-chains are depicted in their crystallographic arrangements for graphical simplicity, note that 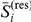 is a measure of the extent of fluctuations among a wide variety of distinct packings. For the results presented in panel (a), I86 (shown in black and circled) was mutated to G. For those of panel (b) E46 (shown in black and circled) was constrained to its crystallographic configuration.

Fig. 1 shows the change in 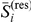 that resulted from a single-residue perturbation. Residues shown in red demonstrated a statistically-significant increase in side-chain variability, while the variability of those shown in blue decreased (see Methods). The perturbations shown, a mutation of isoleucine to glycine at position 86 (Fig. 1(a)) and a constraint of the glutamate in position 46 to its crystalline configuration (Fig. 1(b)), were chosen to demonstrate the types of changes possible. (A comparison to the previously studied P27A [32] was not possible, since neither proline nor alanine residues have rotatable side-chains in our model.) Surprisingly, removing the isoleucine side-chain at position 86 (circled in Fig. 1(a)) not only affected the local entropy of a few neighboring residues, but also altered the side-chain variability of residues much farther from the site of mutation. Motions of even distant residues must therefore be linked to those of residue 86. Because the interaction potentials in our model are short in range, the changes in fluxionality that resulted from this mutation must propagate through neighboring residues to those farther away. Fig. 1(b) shows analogous changes that resulted when a residue, E46 (circled), is merely frozen into its crystalline conformation. Such a reduction in motion of one side-chain might be expected to result in the increased variability of its nearest neighbors. However, we found that even so subtle a constraint resulted in unexpected and wide-spread changes in the side-chain fluctuations. Some residues near the frozen amino acid even became slightly *more* constrained while the variability of a few residues farther away increased. A similar effect was observed in NMR experiments upon ligand-binding in stromelysin 1, where the few residues participating in strong interactions with the ligand lost mobility, but the order parameters of those farther away actually decreased upon binding, indicating an increase in their entropy [36]. It was suggested that the increased fluctuations far from the binding site may counter the loss of entropy at the binding site itself and therefore assist in modulating the thermodynamics of binding.

The changes in side-chain statistics we observed as a result of these single residue perturbations are not readily intuited. Increasing or decreasing disorder at one site may result in the same or opposite effect in other regions of the folded protein, and the effects cannot be easily predicted from the spatial arrangement of the residues.

### Correlated fluctuations result from several types of interactions and persist throughout protein

Correlated fluctuations within the folded protein are commonly quantified using Pearson correlation coefficients [14]. Despite their limitations in detecting nonlinear correlations and correlations between the motions of particles moving orthogonally to one another [37], Pearson coefficients have yielded important information regarding correlated motions. These coefficients are most appropriate for backbone motions as these motions are expected to be correlated in similar directions and to be linear in nature due to the stiffness and collective motions of various secondary structural elements [14]. However, in a study analyzing the results of molecular dynamics simulations of protein G and lysozyme, a generalized correlation measurement based on mutual information was able to detect significantly more correlation than the Pearson coefficient [37]. Side-chain motions are even more likely to fall outside the purview of the Pearson coefficient, dominated as they are by dihedral angle rotations. A parameter based on mutual information is able to provide a more robust measurement of correlated side-chain fluctuations [37], and can be readily derived from simulation data in a similar way to the entropies calculated in the preceding section. We therefore chose to consider the mutual information associated with each pair of residues within a folded protein.

Pairwise mutual information is a measure of the correlation between two random variables. In our case it reports on the degree of correlation between the rotameric state populations of two residues. The mutual information 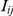 between residues 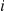 and 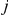 can be calculated as
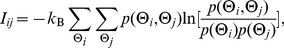
(2)where 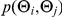 denotes the probability of each of the 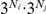 joint states of residues 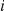 and 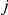, and 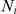 is the number of rotatable 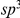-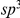 hybridized bonds in residue 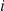. After rearranging Eq. 2 and substituting in Eq. 1, this becomes
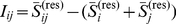
(3)where 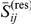 is the Gibbs entropy associated with the discrete rotameric states for residues 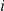 and 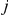 considered jointly. Thus when the fluctuations of the two residues are completely independent of one another, 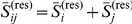 and 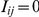. However, when the residues are correlated, their entropies are inseparable, and 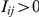.

One difficult feature of mutual information is that a numerically-calculated estimate of two completely uncorrelated variables only approaches zero at the limit of infinite sampling. For any finite sampling, a small amount of spurious mutual information will be observed, regardless of the actual coupling between the two variables [38]. When calculating 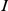 numerically, this inherent bias in the noise must be accounted for in order to determine the mutual information's statistical significance. We used two approaches to address this bias. In the first, we subtracted out the expected spurious mutual information to estimate the true amount of correlation between the two variables. The resulting excess mutual information, 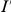, between residues 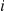 and 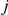 is defined as
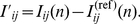
(4)

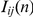
 is the numerically-calculated mutual information measured over a finite sampling period consisting of 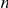 MC steps. 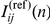 is the same measurement, but this time computed within a non-interacting reference state, where no correlations are possible (see Methods for details). 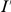 is then an estimate of the mutual information of the infinitely-sampled ensemble. In the second approach, we focused on the robustness of the mutual information measurement, calculating its signal-to-noise ratio, 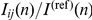.

**Figure 2:**
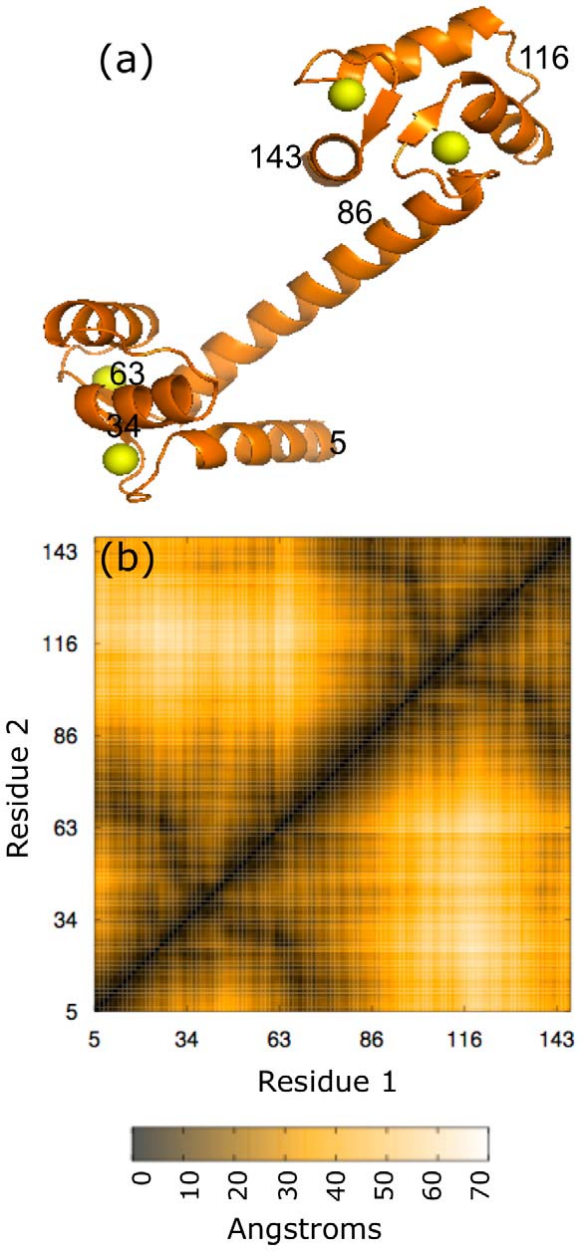
Structural representations of extended crystalline calmodulin. The crystal structure (a) and contact map (b) of calcium-bound calmodulin (3cln [39]). The calcium ions are shown in yellow, and several residues are labeled in both panels for reference. The distance between each pair of 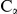 atoms is indicated by color (see scale bar) in (b), where 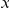
- and 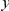-axes run over residue labels. The residue labeling corresponds to the full sequence, however residues that do not possess torsional degrees of freedom in our model (A, G, P, and all residues bound to the calcium ions) are excluded from the contact map.

**Figure 3:**
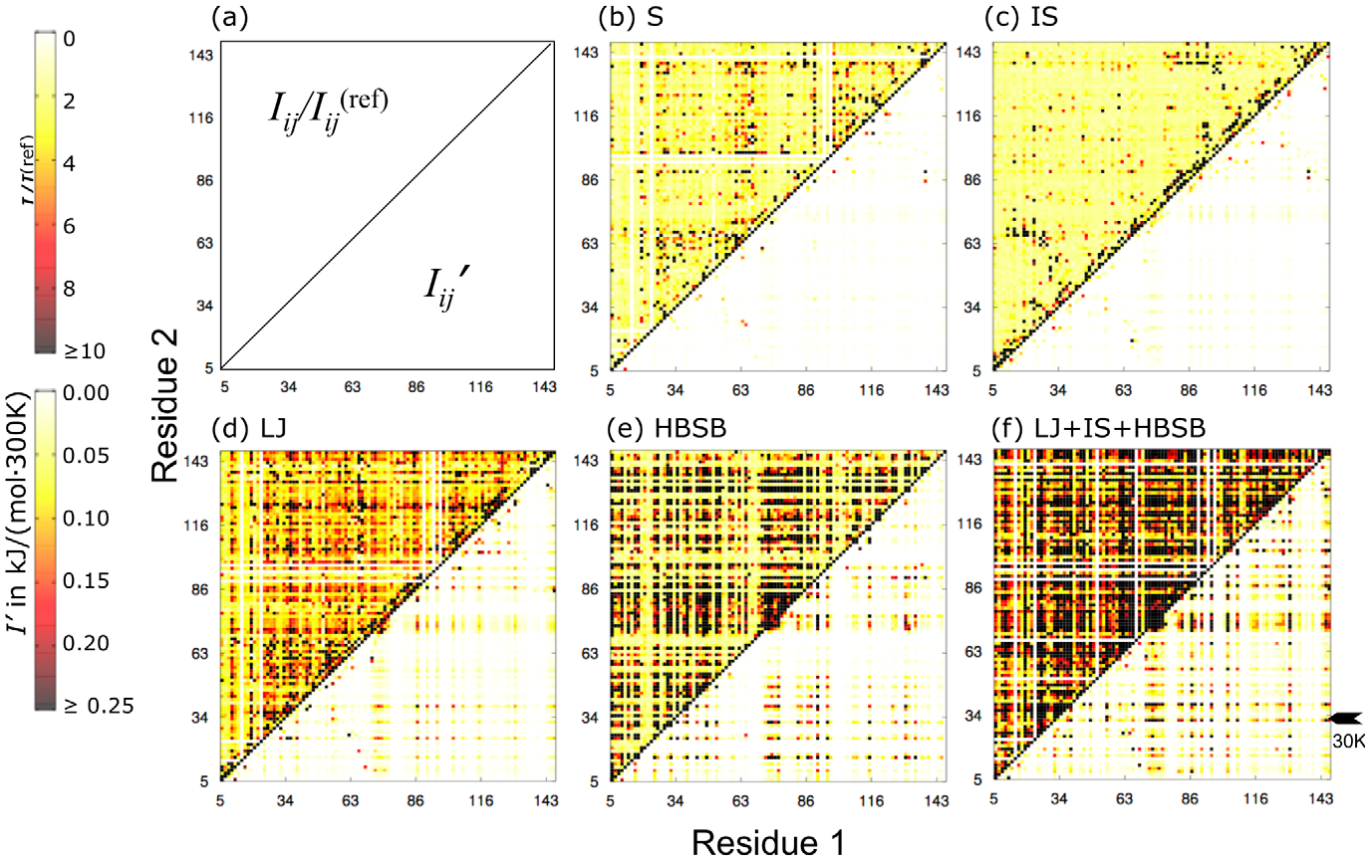
Mutual information of residue pairs in calmodulin. The mutual information, 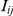, associated with side-chain fluctuations of residue pairs in calmodulin. Plots (b)–(f) display the mutual information signal∶noise ratio, 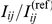 (upper left triangles) and the excess mutual information 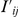 (lower right triangles), as indicated in (a). The 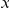
- and 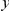-axes run over labels, 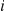 and 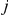 respectively, of residues in the amino acid sequence, excluding those lacking rotameric freedom in our model. Scale bars for the signal∶noise ratio and the excess mutual information are presented on the top and bottom left, respectively. Results are shown for the following combinations of interactions: (b) repulsive sterics (S), (c) implicit solvent (IS) (d) Lennard-Jones (LJ) interaction comprising repulsive sterics and van der Waals attractions, (e) hydrogen bonding and salt-bridges (HBSB), and (f) the full potential (LJ+HBSB+IS). Residue 30K, which we scrutinize in detail later (see Fig. 5), is highlighted in (f) for reference.

The extended structure of calmodulin (3cln [39]), as shown in Fig. 2(a), provides an exemplary test case for examining how side-chain fluctuations are correlated within the folded protein. Although in solution this chain collapses, the structure of the crystal is extended, featuring two globular regions connected by an extended 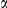-helix. Any information shared between the two lobes must pass through this extended 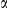-helix, since the pairwise interactions in our model largely decay by 7 Å. We calculated the pairwise excess mutual information, 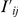, for all residue pairs 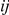 in 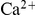-bound calmodulin, as well as the ratio of 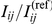 in order to gauge the significance of the measured correlations. Both quantities are shown in Fig. 3 as functions of the residues' position along the backbone. For reference, we present in Fig. 2(b) the spatial distance between residues in the native structure as a function of the same indices. Panels (b)–(e) of Fig. 3 indicate mutual information resulting from various interaction types considered in isolation. Panel (f) gives results for the full model.

Different types of inter-atomic interactions in our model gave rise to different patterns of correlated fluctuations. In Fig. 3(b), correlations that result solely from steric repulsions are shown. While the signature of calmodulin's 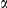-helical structure can be clearly seen along the diagonal, where residues 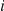 and 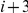 or 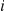 and 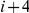 are often highly correlated, many other residue pairs appear significantly correlated as well, even those that are spatially distant. In Fig. 3(c), the correlations that result from the implicit solvent alone are shown. These correlations are more limited, restricted almost completely to residues that are nearby in space, as can be seen when comparing Fig. 3(c) to Fig. 2(b). Again the 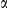-helical residues display appreciable correlation, even more than that resulting from the repulsive sterics, as might be expected from their high degree of solvent exposure. The correlations that result from considering van der Waals attractions along with the repulsive sterics is shown in Fig. 3(d). While the correlations along the 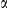-helix remain strong, many other correlations emerge as a result of these attractions. Hydrogen bonding and salt bridge interactions, taken alone, generate highly significant correlations throughout the entire structure (see Fig. 3(e)), which appear remarkably insensitive to spatial distance. Since only a subset of the residues participate in such interactions, the fluctuations of the remaining residues are completely uncorrelated in this restricted version of our model. The full potential, used to generate the data in Fig. 3(f), results in both the most significant signal-to-noise ratios and the largest excess mutual information values, indicating a large degree of correlation that spans the full range of inter-residue distances while retaining features of the dominant 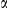-helical structure.

**Figure 4:**
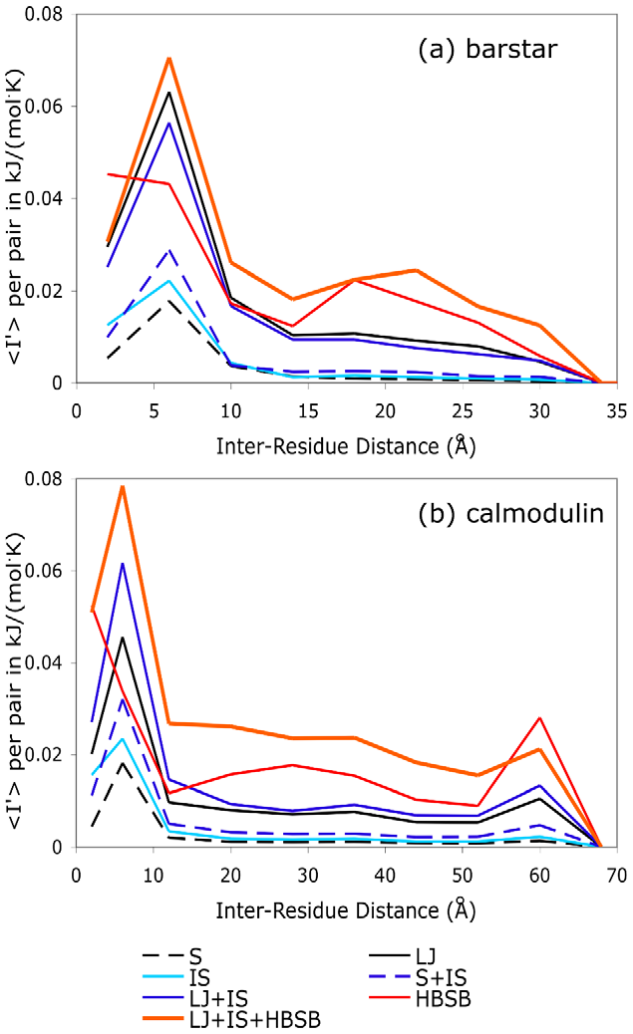
Distance dependence of mutual information in barstar and calmodulin. Average excess mutual information 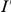 as a function of distance between 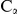 atoms. For both (a) barstar and (b) calmodulin, we summed the values of 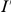 for all residue pairs within several inter-residue distance ranges and then divided by the number of such pairs. Results are shown for various subsets of atomic interactions: S indicates repulsive sterics, IS indicates implicit solvent, LJ indicates the Lennard-Jones interaction, and HBSB indicates the hydrogen bonding and salt-bridge interactions. See Methods for binning details.

To further explore how different interactions give rise to long-range correlations in both a small globular protein as well as the extended calmodulin structure, we calculated the average excess mutual information per residue pair for all residue pairs in calmodulin and barstar, resolved by the spatial inter-residue distance between 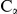 atoms. (See Fig. 4.) In both proteins, steric repulsions alone give rise to small, but significant, correlations that persist across the entire protein structure. The same is true for the implicit solvent interactions and their combination, S+IS. However, much larger correlations emerge when van der Waals attractions are considered in addition to the steric repulsions. Hydrogen bonding and salt bridge interactions are clearly the most correlating types of interactions considered. However, the full potential, which combines all these interaction types, results in the largest overall correlation.

An additional feature within these plots deserves mention; in both proteins, correlation is at a maximum around 6 Å for all subsets of interactions excepting hydrogen bonding and salt bridges. This short-distance peak indicates that residue pairs adjacent in the amino acid sequence (whose 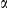-carbons are separated by 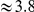 Å) do not interact as strongly on average as do residue pairs that are positioned slightly farther apart. In 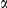-helices, neighboring residues point in different directions and, while still likely to interact with their sequential nearest neighbor, are more likely to interact strongly with their 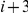 and 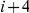 neighbors. In 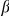-sheets, however, residues alternately point towards different faces of the sheet, so that the side-chains on residues 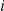 and 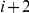 are much more likely to interact with one another than do those on 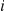 and 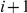. Residues influenced only by hydrogen bonding and salt bridge interactions, when artificially freed of the steric constraints that would keep them from collapsing back on themselves, still correlate most strongly with their nearest neighbors.

Substantial long-range correlation is seen throughout both barstar and calmodulin. Moreover, the fact that so many subsets of the full potential independently give rise to long-range correlations suggests that correlated side-chain fluctuations should be a robust characteristic of most protein sequences and nearly any globular fold.

### Correlated side-chain motions can propagate changes in fluctuations over 50 Å

Through correlated side-chain fluctuations, local perturbations to a protein (e.g., due to small ligand binding) could in principle be transmitted over substantial distances. We scrutinized this possibility by examining the consequences of mutating a single residue in calmodulin. Such a mechanism of communication was described earlier for barstar (see Fig. 1), whose size limited our conclusions to distances of less than 30 Å. Calmodulin, in its extended structure, provides a better test of the ability of side-chain correlations to transmit information over long distances.

**Figure 5:**
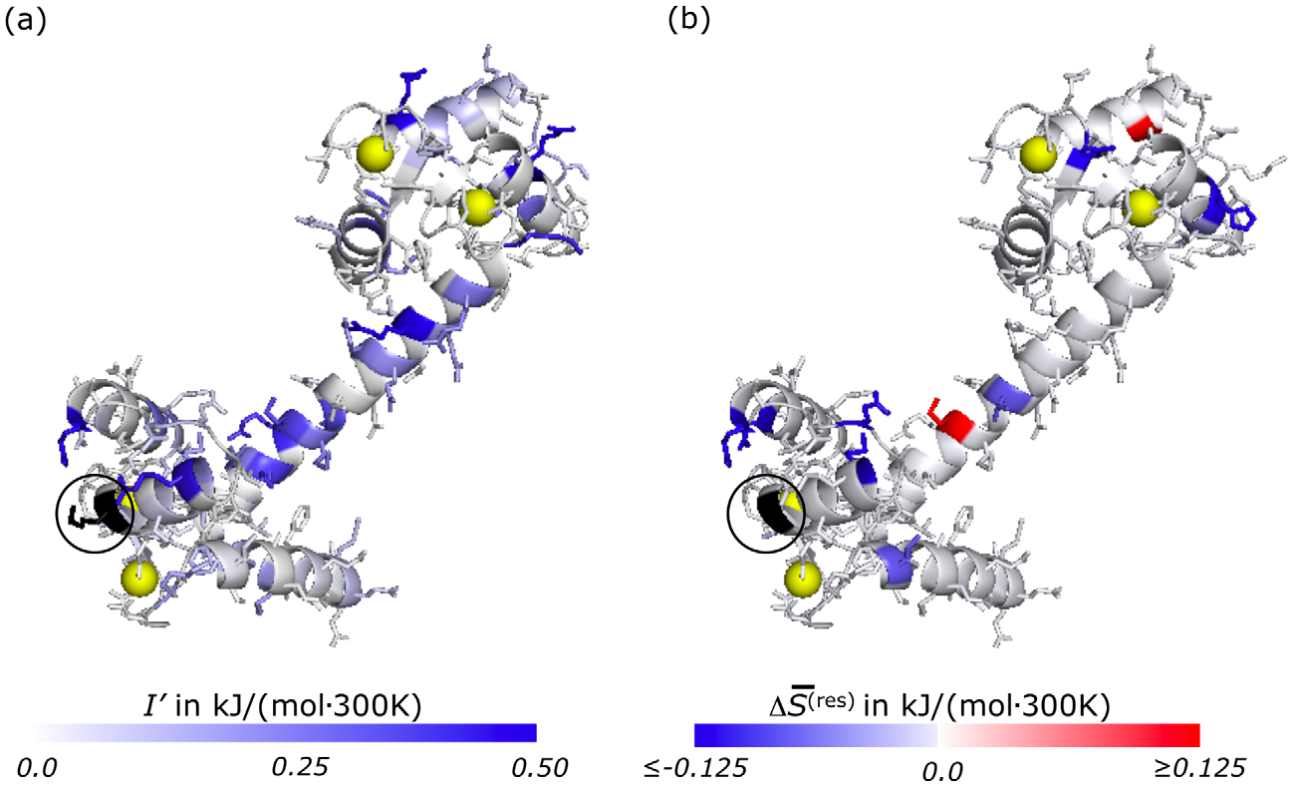
Correlation between residue 30 and other residues in calmodulin. The extent of correlation between residue 30 (shown in black and circled) and all other side-chains in calmodulin (3cln [39]) is shown here. In (a) each residue 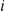 is colored according to the magnitude of its excess mutual information 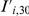 with 30K (see left scale bar and Fig. 3). Coloring in (b) indicates the change 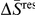 in each residue's side chain entropy effected by the mutation K30G. Here, red represents increased entropy and blue decreased entropy (see right scale bar). See Methods for details.

We focused this analysis on correlated fluctuations involving one particular residue in calmodulin, 30K, which we observed to be significantly correlated with several other residues (see Fig. 3(f)). In Fig. 5(a), 30K is colored black, while the pairwise mutual information between this side-chain and all others is indicated in bluescale.

Appreciable correlations are apparent throughout the lower globular region near residue 30. The correlations become stronger within the spatially-constricted alpha-helical bridge and spread out again and weaken in the far lobe. To determine whether these correlations could transmit structural and dynamical information over significant distances, we mutated residue 30K to glycine. The resulting change in 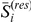 for each residue 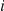 is shown in Fig. 5(b). A significant decrease in entropy was detected in some neighboring residues, while both increases and decreases in entropy were found for residues farther from the mutation site. Although unexpected, the reduction in entropy at residue 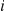 resulting from the removal of a neighboring bulky residue can be readily explained if that nearby mutation results in the loss of a potential hydrogen bonding partner for residue 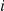. Such a loss can result in the probability associated with the hydrogen-bonding subset of residue 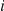
's configurations being greatly reduced.

We found statistically significant changes in entropy even in the globular region opposite that of residue 30. Thus we conclude that side-chain fluctuations alone can reliably propagate the effect of a single point mutation over at least 50 Å.

When comparing Fig. 5(a) to Fig. 5(b), it is clear that some, but not all, of the strongly correlated residues in the wild-type calmodulin experience detectable changes in their side-chain variability as a result of this particular mutation. Even some residues that are minimally correlated with residue 30K show significant shifts in their side-chain statistics. Although the mutual information can tell us a great deal about the degree of correlation between two side-chains in our model, it is not a discriminating predictor of changes in side-chain variability upon mutation. The discrepancies are likely due to the fact that our calculation of mutual information lacks contributions from correlated intra-rotameric fluctuations, which are still able to convey information in our model and will therefore influence the detected changes upon side-chain mutation. Furthermore, observing the statistically significant changes in Fig. 5(b) requires a great deal of sampling – were more sampling feasible, additional changes would likely be detected.

### Magnitude of side-chain correlations is substantial

If the side-chain motions of a protein's 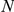 different residues were negligibly correlated, then the total entropy 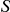 associated with transitions among distinct rotameric states could be calculated as a simple sum of single-residue contributions, 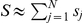. The excess mutual information, summed over all residue pairs, provides a rough measure of the error in such a mean-field estimate. Correspondingly, the quantity 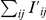 characterizes the global thermodynamic significance of inter-residue correlations. For crystalline barstar modeled with the full potential, 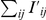 is calculated to be 72 kJ/(mol

300 K). The higher-order correlations expected in such a dense environment [40] (see Fig. 3 where a single residue is often significantly correlated to several others) make this value an overestimate of the total correlation. Even so, its magnitude is noteworthy. In addition, while allowing intra-rotameric fluctuations, this calculation neglects their contribution to the total correlation, which were found to be essential in reproducing the calorimetric 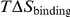 of calmodulin with its ligands in [21] and are likely to be substantial.

### Long-range correlations are present within several different backbone models of folded barstar

The rigidity of the peptide backbone in these calculations justifies to some extent our schematic model of side chain interactions: For our purposes the potential energy function need not resolve subtle thermodynamic differences among diverse chain conformations, but instead serves to establish basic length and energy scales for rearrangements within the native state's basin of attraction.

In considering the biological relevance of our results, backbone rigidity is in part justified empirically by the observation that only weak correlations exist between backbone NMR order parameters, 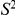, and their associated side-chain order parameters, 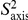 [41]. This weak correlation is likely due to the fact that side-chain and backbone fluctuations largely occur on different time-scales [42], with typical side-chain fluctuations ranging from picoseconds to nanoseconds, while typical collective backbone fluctuations range from nanoseconds to seconds and longer.

**Figure 6:**
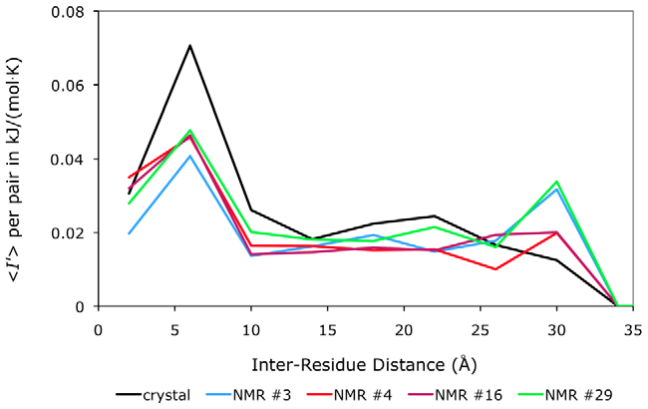
Distance dependence of mutual information in different NMR models of barstar. The average excess mutual information 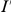 per residue pair is plotted here for various atomic interactions, binned according to the 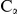-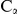 inter-residue distance, for the crystal structure (1a19 [47]) and four NMR model structures (1btb [43]) of barstar, using the full LJ+IS+HBSB potential. See Methods for details.

However, it is important to assess how variations in backbone configuration of the folded protein might influence the side-chain correlations we have calculated. Toward this end we examined four different structural models from an NMR structure of barstar (1btb [43]). These four conformations were chosen to represent the range of models included in the NMR structure (see Methods). In each case plots of 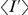 per pair vs. inter-residue distance for the full potential closely resemble results for the crystal structure (see Fig. 6). Since the statistics of side-chain rotations in a fluctuating backbone environment can be rigorously decomposed into contributions from sub-ensembles in which the backbone is held fixed, the consistent nature of the observed long-range correlation from one backbone structure to another establishes their robustness to typical backbone motions.

Larger backbone fluctuations, however, such as partial unfolding events or the motions of hinged regions, are certain to disrupt many of these correlations and may limit their role in conveying allosteric information. In particular those correlations arising from contact between residues that are spatially proximal, but distant within the protein's amino acid sequence, will attenuate as backbone motions carry them away from one another. However, correlations between residues linked through a path of sequential neighbors, such as those observed along the central 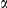-helix of crystalline calmodulin in Fig. 5, may persist. As a result, some information may continue to be transmitted through side-chain fluctuations even after significant backbone rearrangements, as long as the secondary structure, which is responsible for many of the observed correlations between sequential neighbors (see Fig. 3(f)), remains intact.

### Amino acids displayed different propensities towards correlated side-chain motion

**Figure 7:**
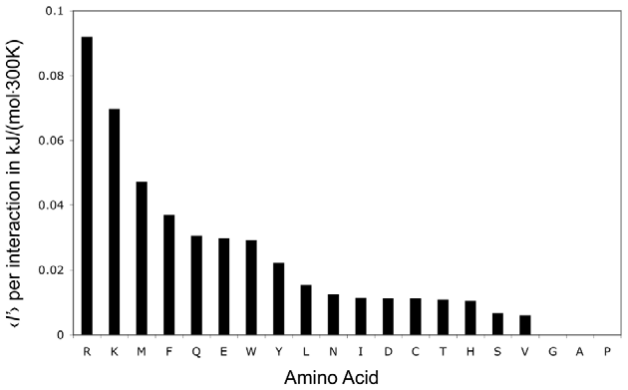
Mutual information by residue type. The average excess mutual information per interaction, 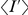, for all twenty amino acids. In each case data was pooled from all applicable pairs of fluctuating residues within a set of twelve small globular proteins (see Methods).

In addition to scrutinizing the effect of different types of atomic interactions, we also examined how a protein's amino acid composition might contribute to stronger or weaker correlations among its side-chain fluctuations. To do so, we took a set of twelve small globular proteins with different sequences and folds (see Methods) and calculated the average excess mutual information per pairwise interaction for each amino acid across the entire set of proteins. The result is plotted in Fig. 7.

In general, amino acids with the most 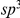-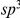 hybridized rotatable bonds resulted in the largest 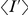 values. The amino acid arginine is clearly the most strongly correlated residue, followed closely by lysine. While both of these amino acids have four rotatable bonds, arginine is considerably bulkier than lysine, with more potential hydrogen bonding partners. In addition, arginine has been found to take on fewer alternate rotameric states in simulations of folded proteins than lysine [44].

Similarly, the bulky aromatics (Phe, Tyr, Trp) were more correlating than their single 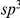-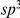 hybridized rotamer would indicate, while isoleucine and leucine are both much less correlating than the other residues with two rotatable bonds: glutamine and glutamate. Glycine, alanine, and proline all have 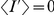, since they possess no rotatable bonds in the model.

### Changes in NMR-derived order parameters do not compare well to calculated changes in side-chain entropies

Recent NMR measurements on eglin C provided a good opportunity to compare our results with experimental evidence of wide-spread changes in side-chain fluctuations resulting from small perturbations [9], [30]. In this work, a series of valine residues were mutated to alanine at various positions in eglin C, a small globular protein with a relatively static backbone, and the resulting changes in the order parameters of side-chain methyl groups were measured. The changes in the NMR-measured order parameters were in many cases quite low; the majority of the statistically significant changes were less than 0.05 (order parameters range from 0.0 for a completely disordered vector to 1.0 for a completely ordered one), with only a few residues showing changes greater than 0.1 [9], [30]. The magnitude of these NMR-measured changes combined with the significant statistical errors in our calculations (the average standard deviation was 0.02) rendered such a comparison difficult. In the cases where we could resolve the changes in our MC-calculated order parameters enough to make a meaningful comparison to the experiments we found little correspondence between our data and the NMR measurements.

NMR order parameters are expected to underestimate the full range of side-chain motion, as they neglect motion slower than the tumbling time of the molecule, and recent work demonstrates that such motion can be substantial [45], [46]. Similarly, our calculation is also expected to underestimate the full range of motion accessible to the side-chains due to the fixed backbone, which we observed previously to be particularly problematic in calculating methyl group order parameters for alanines [21]. As a result, a poor correspondence between our calculated methyl-group order parameters and those derived from NMR relaxation experiments, in particular those involving mutations to alanine, is perhaps to be expected. In past work we nevertheless demonstrated a clear correspondence to the measured NMR order parameters for wild-type eglin C using the same computational approach [21]. Even if the model is not sufficiently accurate or detailed to make quantitative predictions for altered side-chain fluctuations in the specific case of eglin C, the conclusions we draw here for long-range correlations among side-chain fluctuations should be pertinent to the biophysics of folded proteins in general.

## Discussion

The propagation of information across long distances within the folded protein is of great importance in allosteric regulation. While backbone structural changes and fluctuations have long been studied as the bearer of this information, correlated side-chain fluctuations provide potential information conductance pathways that have often been overlooked. In this work we have examined these side-chain correlations and found that changes in fluctuations in response to even single residue perturbations, such as a point mutation or residue immobilization, are statistically significant, widely distributed, and not easily intuited from the protein's sequence or structure. The correlations emerge independently from several different sources: steric repulsions, solvation, and hydrogen bonding and salt bridges. Together, these interactions give rise to robust, statistically-significant correlations that persist across the entire spatial extent of both barstar and calmodulin.

In the calculation of the mutual information values, we model the protein's backbone as a rigid structure, enabling us to investigate the degree of correlation among side-chain fluctuations alone. An understanding of the role of backbone fluctuations is necessary, however, to judge the biological relevance of these observed correlations. Interestingly, we found that significant correlations are present among the side-chains of barstar in several different backbone structures, which collectively represent the range of its typical conformational fluctuations in solution. Moreover, since the time scales characteristic of backbone and side-chain motions are likely well separated in many cases, communication via side-chain rearrangements as we have described may often occur on an effectively static backbone. However, upon larger backbone rearrangement, such as hinge motions or partial unfolding events, a significant fraction of the observed side-chain fluctuations are likely to decouple, and thus the role of side-chain correlations in allosteric regulation where large backbone rearrangements are known to occur may be limited.

We also utilized a simple implicit solvent model to account for the solvent's mean field effect on the protein. While this approach allows us to focus directly on the protein's degrees of freedom, it neglects solvent fluctuations. Physically realistic solvent fluctuations are likely to influence side-chain fluctuations in the same way that side-chain fluctuations influence one another, and the reverse is also true. This potential for correlation between solvent and side-chain fluctuations suggests that fluctuations of the solvent shell may also convey information from one site on the protein to another. Indeed, we observed that even the mean field effect of solvation mediates the correlation of side-chain fluctuations, as seen in Fig. 3(c). However, more and stronger correlations were observed to arise from hydrogen-bonding and salt bridge interactions (see Figs. 3(e) & 4), and it is largely through these very effects that the solvent molecules would influence the side-chains. While we are not able to explore the resulting implications in our current work, the demonstration of allosteric effects mediated through solvent fluctuations would be quite intriguing.

Finally, it is important to note that the magnitude of the correlations measured here is only a fraction of the total magnitude possible among the side-chains, since correlated fluctuations within each individual rotameric well, which are not included in our correlation metric, are sure to contribute significantly, perhaps even to a greater degree, to the overall amount of correlation. Even so, correlations amongst inter-rotameric fluctuations alone reveal much about the way side-chain fluctuations give rise to long-range correlations within the folded protein. The role of these robustly correlated side-chain fluctuations in allosteric regulation should be considered further.

## Methods

### Model

Our model is defined by an energy function that depends on the atomic coordinates of the protein's residues. The only degrees of freedom in our model are the dihedral angles 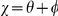, where 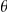 represents the ideal angle of each rotameric state taken from an empirical rotamer library [33], and 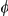 describes torsional variations within the potential energy well of each rotamer. Thus the energy function depends on the full set of 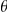 and 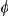 values (denoted 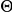 and 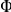) for all residues,
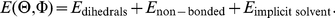
(5)


The potential 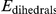 is piecewise quadratic in 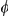, biasing dihedral angles towards their ideal 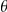 values; 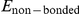 includes a Lennard-Jones function that governs both repulsive sterics (with a hard-sphere cutoff at 0.75 times the van der Waals radius) and attractive van der Waals interactions, as well as hydrogen-bonding and salt-bridge terms; and 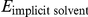 accounts for solvation using an approach based on the solvent-accessible surface area. A detailed description of the model is given in [21].

### Structures

The analysis outlined in this paper requires a good structural model of the protein considered. For barstar, we use a crystal structure of the mutant C82A at 2.8 Å resolution (1a19 [47]), except for the analysis in Fig. 6 using different NMR structural models, where the structure 1btb [43] was used. In this case four models (numbers 3, 4, 16, and 29) were chosen to represent the structural variety within the full set of NMR models, as assessed by the RMSD values calculated between all possible pairwise combinations of the four models and compared to those of the full ensemble.

For calmodulin, a crystal structure at 2.2 Å was used (3cln [39]). In our calculations the positions of calcium ions and the side-chains bound to them were held fixed. The results in Fig. 7 also included the following proteins: eglin c (1cse [48]), GB3 (1igd [49]), protein L (1hz6 [50]), PYP (1f9i [51]), PZD2 (1r6j [52]), SH2 (1d1z [53]), CspA (1mjc [54]), ubiquitin (1ubq [55]), and tenascin (1ten [56]).

### Sampling

Side chain configurations were sampled from the Boltzmann distribution using Metropolis Monte Carlo techniques. In order to calculate the highly converged measurements of 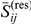 needed to produce the results shown in Figs. 1 & 5(b), additional adaptive umbrella sampling techniques and biased sampling procedures were utilized [21], [57]. Averages at 300 K were finally constructed from such calculations by summing results for different energies with appropriate statistical weights.

### Statistical tests

#### Significance of 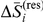



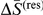
 values in Figs. 1 & 5(b) were color-coded only if they passed Student's t-test for significance at the 90% level, using the two-sided Student's t statistic. Mean 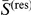 values were calculated using a Wang-Landau [57] bias for five sets of five trials each, first for the unperturbed wild-type protein and then for the same protein perturbed either by freezing or by mutating one of its side-chains. Averages and standard deviations were calculated across the five sets and compared to determine the significance. For Fig. 1, trials ran for 200,000 MC sweeps, whereas for Fig. 5(b), trials ran for 50,000 MC sweeps.

#### Significance of mutual information

The calculation of both the excess mutual information 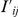 and the signal-to-noise ratio of the mutual information 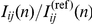 requires an estimate of the spurious mutual information 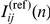 resulting from finite sampling (see Eq. 4). We constructed 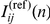 by sampling rotameric states from a non-interacting reference system defined by the energy function
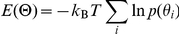
Here, 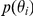 is the probability of observing rotamer state 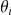 in the fully interacting model. By construction, these single-residue distributions are then identical in the reference system, 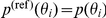. Rotameric fluctuations of distinct residues, however, are statistically independent in the reference system, 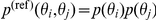, so that 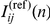 vanishes in the limit of complete sampling, 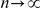.

For each interacting trial run, the probabilities 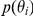 associated with each rotameric state were recorded and used to bias its corresponding non-interacting reference run. Excess mutual information values and signal-to-noise ratios were then calculated independently for each pair of interacting and non-interacting, but biased, trial runs. Presented results are averages of these 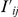 and 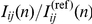 values.

For the results shown in Figs. 3, 4, 5(a), & 6, 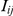 was calculated in five trial runs of 50,000 MC sweeps initiated from randomly-chosen side-chain configurations. Subsequently, 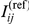 was computed from five independent runs of a biased, noninteracting reference system, also initiated from randomly-chosen side-chain configurations. As described above, the lengths and biases of these reference runs were chosen to produce samples equivalent in size and in single-rotamer distribution to the number and distribution of sterically valid configurations generated in the corresponding simulation of the interacting system. The signal∶noise ratio and the excess mutual information were calculated independently for each trial; averages over those trials are presented in the figures. The results shown in Fig. 5(a) and Fig. 6 were calculated using the full LJ+IS+HBSB potential.

For Figs. 4 & 6, the above results were collected into inter-residue distance bins of 4 Å wide for barstar and 8 Å wide for calmodulin, except for the first two bins which were kept 4 Å wide in order to highlight the peak at short distances of 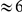 Å.

All images were made using MacPyMOL [58].

## References

[1] Barford D, Johnson LN (1989). The allosteric transition of glycogen phosphorylase.. Nature.

[2] Ottemann KM, Xiao W, Shin YK, Koshland DE (1999). A piston model for transmembrane signaling of the aspartate receptor.. Science.

[3] Perutz MF (1970). Stereochemistry of cooperative effects in haemoglobin.. Nature.

[4] Volkman BF, Lipson D, Wemmer DE, Kern D (2001). Two-state allosteric behavior in a singledomain signaling protein.. Science.

[5] Cooper A, Dryden DT (1984). Allostery without conformational change. a plausible model.. Eur Biophys J.

[6] Popovych N, Sun S, Ebright RH, Kalodimos CG (2006). Dynamically driven protein allostery.. Nat Struct Mol Biol.

[7] Tzeng SR, Kalodimos CG (2009). Dynamic activation of an allosteric regulatory protein.. Nature.

[8] Gunasekaran K, Ma B, Nussinov R (2004). Is allostery an intrinsic property of all dynamic proteins?. Proteins.

[9] Clarkson MW, Gilmore SA, Edgell MH, Lee AL (2006). Dynamic coupling and allosteric behavior in a nonallosteric protein.. Biochemistry.

[10] Petit CM, Zhang J, Sapienza PJ, Fuentes EJ, Lee AL (2009). Hidden dynamic allostery in a pdz domain.. Proc Natl Acad Sci USA.

[11] Mayer KL, Earley MR, Gupta S, Pichumani K, Regan L (2003). Covariation of backbone motion throughout a small protein domain.. Nat Struct Biol.

[12] Bouvignies G, Bernadó P, Meier S, Cho K, Grzesiek S (2005). Identification of slow correlated motions in proteins using residual dipolar and hydrogen-bond scalar couplings.. Proc Natl Acad Sci USA.

[13] Lange OF, Grubmüller H, de Groot BL (2005). Molecular dynamics simulations of protein g challenge nmr-derived correlated backbone motions.. Angew Chem Int Ed.

[14] Ichiye T, Karplus M (1991). Collective motions in proteins: a covariance analysis of atomic fluctuations in molecular dynamics and normal mode simulations.. Proteins.

[15] Henzler-Wildman KA, Lei M, Thai V, Kerns SJ, Karplus M (2007). A hierarchy of timescales in protein dynamics is linked to enzyme catalysis.. Nature.

[16] Ota N, Agard DA (2005). Intramolecular signaling pathways revealed by modeling anisotropic thermal diffusion.. J Mol Biol.

[17] Sharp K, Skinner JJ (2006). Pump-probe molecular dynamics as a tool for studying protein motion and long range coupling.. Proteins.

[18] Igumenova TI, Frederick KK, Wand AJ (2006). Characterization of the fast dynamics of protein amino acid side chains using nmr relaxation in solution.. Chem Rev.

[19] Lang PT, Ng HL, Fraser JS, Corn JE, Echols N (2010). Automated electron-density sampling reveals widespread conformational polymorphism in proteins.. Protein Sci.

[20] Frederick KK, Marlow MS, Valentine KG, Wand AJ (2007). Conformational entropy in molecular recognition by proteins.. Nature.

[21] DuBay KH, Geissler PL (2009). Calculation of proteins' total side-chain torsional entropy and its influence on protein-ligand interactions.. J Mol Biol.

[22] Marlow MS, Dogan J, Frederick KK, Valentine KG, Wand AJ (2010). The role of conformational entropy in molecular recognition by calmodulin.. Nat Chem Biol.

[23] Millet O, Mittermaier A, Baker D, Kay LE (2003). The effects of mutations on motions of side-chains in protein l studied by 2 h nmr dynamics and scalar couplings.. J Mol Biol.

[24] Dhulesia A, Gsponer J, Vendruscolo M (2008). Mapping of two networks of residues that exhibit structural and dynamical changes upon binding in a pdz domain protein.. J Am Chem Soc.

[25] Fersht A (1999). Structure and Mechanism in Protein Science.

[26] Lockless SW, Ranganathan R (1999). Evolutionarily conserved pathways of energetic connectivity in protein families.. Science.

[27] Süel GM, Lockless SW, Wall MA, Ranganathan R (2003). Evolutionarily conserved networks of residues mediate allosteric communication in proteins.. Nat Struct Biol.

[28] Noivirt O, Eisenstein M, Horovitz A (2005). Detection and reduction of evolutionary noise in correlated mutation analysis.. Protein Eng Des Sel.

[29] Zídek L, Novotny MV, Stone MJ (1999). Increased protein backbone conformational entropy upon hydrophobic ligand binding.. Nat Struct Biol.

[30] Clarkson MW, Lee AL (2004). Long-range dynamic effects of point mutations propagate through side chains in the serine protease inhibitor eglin c.. Biochemistry.

[31] Wong KB, Daggett V (1998). Barstar has a highly dynamic hydrophobic core: evidence from molecular dynamics simulations and nuclear magnetic resonance relaxation data.. Biochemistry.

[32] Li H, Frieden C (2007). Comparison of c40/82a and p27a c40/82a barstar mutants using 19f nmr.. Biochemistry.

[33] Lovell SC, Word JM, Richardson JS, Richardson DC (2000). The penultimate rotamer library.. Proteins.

[34] Kussell E, Shimada J, Shakhnovich EI (2001). Excluded volume in protein side-chain packing.. J Mol Biol.

[35] Shetty RP, Bakker PIWD, DePristo MA, Blundell TL (2003). Advantages of fine-grained side chain conformer libraries.. Protein Eng.

[36] Arumugam S, Gao G, Patton BL, Semenchenko V, Brew K (2003). Increased backbone mobility in beta-barrel enhances entropy gain driving binding of n-timp-1 to mmp-3.. J Mol Biol.

[37] Lange OF, Grubmüller H (2006). Generalized correlation for biomolecular dynamics.. Proteins.

[38] Roulston MS (1999). Estimating the errors on measured entropy and mutual information.. Physica D.

[39] Babu YS, Bugg CE, Cook WJ (1988). Structure of calmodulin refined at 2.2 a resolution.. J Mol Biol.

[40] Killian BJ, Kravitz JY, Gilson MK (2007). Extraction of configurational entropy from molecular simulations via an expansion approximation.. J Chem Phys.

[41] Mittermaier A, Kay LE, Forman-Kay JD (1999). Analysis of deuterium relaxation-derived methyl axis order parameters and correlation with local structure.. J Biomol NMR.

[42] Petsko GA, Ringe D (2004). Protein Structure and Function..

[43] Lubienski MJ, Bycroft M, Freund SM, Fersht AR (1994). Three-dimensional solution structure and 13c assignments of barstar using nuclear magnetic resonance spectroscopy.. Biochemistry.

[44] Berezovsky IN, Chen WW, Choi PJ, Shakhnovich EI (2005). Entropic stabilization of proteins and its proteomic consequences.. PLoS Comput Biol.

[45] Maragakis P, Lindorff-Larsen K, Eastwood MP, Dror RO, Klepeis JL (2008). Microsecond molecular dynamics simulation shows effect of slow loop dynamics on backbone amide order parameters of proteins.. J Phys Chem B.

[46] Farés C, Lakomek NA, Walter KFA, Frank BTC, Meiler J (2009). Accessing ns-micros side chain dynamics in ubiquitin with methyl rdcs.. J Biomol NMR.

[47] Ratnaparkhi GS, Ramachandran S, Udgaonkar JB, Varadarajan R (1998). Discrepancies between the nmr and x-ray structures of uncomplexed barstar: analysis suggests that packing densities of protein structures determined by nmr are unreliable.. Biochemistry.

[48] Bode W, Papamokos E, Musil D (1987). The high-resolution x-ray crystal structure of the complex formed between subtilisin carlsberg and eglin c, an elastase inhibitor from the leech hirudo medicinalis. structural analysis, subtilisin structure and interface geometry.. Eur J Biochem.

[49] Derrick JP, Wigley DB (1994). The third igg-binding domain from streptococcal protein g. an analysis by x-ray crystallography of the structure alone and in a complex with fab.. J Mol Biol.

[50] O'Neill JW, Kim DE, Baker D, Zhang KY (2001). Structures of the b1 domain of protein l from peptostreptococcus magnus with a tyrosine to tryptophan substitution.. Acta Crystallogr D.

[51] Brudler R, Meyer TE, Genick UK, Devanathan S, Woo TT (2000). Coupling of hydrogen bonding to chromophore conformation and function in photoactive yellow protein.. Biochemistry.

[52] Kang BS, Devedjiev Y, Derewenda U, Derewenda ZS (2004). The pdz2 domain of syntenin at ultrahighresolution: bridging the gap between macromolecular and small molecule crystallography.. J Mol Biol.

[53] Poy F, Yaffe MB, Sayos J, Saxena K, Morra M (1999). Crystal structures of the xlp protein sap reveal a class of sh2 domains with extended, phosphotyrosine-independent sequence recognition.. Mol Cell.

[54] Schindelin H, Jiang W, Inouye M, Heinemann U (1994). Crystal structure of cspa, the major cold shock protein of escherichia coli.. P Natl Acad Sci USA.

[55] Vijay-Kumar S, Bugg CE, Cook WJ (1987). Structure of ubiquitin refined at 1.8 a resolution.. J Mol Biol.

[56] Leahy DJ, Hendrickson WA, Aukhil I, Erickson HP (1992). Structure of a fibronectin type iii domain from tenascin phased by mad analysis of the selenomethionyl protein.. Science.

[57] Wang F, Landau DP (2001). Efficient, multiple-range random walk algorithm to calculate the density of states.. Phys Rev Lett.

[58] DeLano W (2007). MacPyMOL: A PyMOL-based Molecular Graphics Application for MacOS X..

